# Tibial plateau fractures: three dimensional fracture mapping and morphologic measurements

**DOI:** 10.1007/s00264-022-05434-w

**Published:** 2022-05-17

**Authors:** Peifeng Yao, Maoqi Gong, Lei Shan, Dong Wang, Yuanming He, Hanzhou Wang, Junlin Zhou

**Affiliations:** 1 Beijing Chao-yang Hospital, Capital Medical University, Gongtinan Road 8#, Beijing, 100020 China; 2 Capital Medical University, Beijing, 100020 China; 3 Beijing Jishuitan Hospital, Beijing, 100020 China

**Keywords:** Tibial plateau fracture, Fracture mapping, Morphology, Classification

## Abstract

**Purpose:**

The injury mechanisms and classifications of tibial plateau fractures (TPFs) are still controversial. The aim of this study is to show 3D fracture mapping of different types of tibial plateau fractures. Moreover, combined with Schatzker and ten-segment classification, we aimed to analyze the injury frequency and characteristics of different segments.

**Methods:**

In total, 346 patients with TPFs treated at level I trauma centres from 2017 to 2021 were reviewed. The CT files of the included cases were typed and categorized. 3D reconstruction of TPFs patients’ CT files were performed using software. All fracture lines were superimposed on the standard model by the software to create TPFs 3D fracture mapping.

**Results:**

This study included 204 male and 142 female patients (average age, 47 years [range, 18 to 83 years]) with a tibial plateau fracture. Using the Schatzker classification, we found 39 type I (11.27%), 103 type II (29.77%), nine type III (2.60%), 71 type IV (20.52%), 52 type V (15.03%), 59 type VI (17.05%) fractures, and 13 others (3.76%). The density areas of fracture lines are mainly located in the ALC and PLC segments (74.3%, 69.1%). In different views, fracture lines of different Schatzker types showed distinct distribution characteristics.

**Conclusions:**

Schatzker classification combined with 3D fracture mapping provides a new presentation of tibial plateau fracture morphology. According to the 3D fracture mapping, different types of TPFs have distinctly different distribution characteristics of fracture lines. There are significant differences between different types of fracture injury segments.

## Introduction

Tibial plateau fractures (TPFs) are a common intra-articular fracture in clinical practice. Because of the complex injury mechanisms and diverse fracture patterns of TPFs, it presented a great challenge to clinical practice [1–4]. Therefore, a better understanding of the distribution of the fracture line and the morphological characteristics of the fracture mass is essential for making treatment decisions.

Previously, plain radiograph-based fracture classification was widely used in the treatment of tibial plateau fractures. With the continuous development of computed tomography (CT), more and more 3D CT-based classification has been reported in the literatures. However, the most common Schatzker classification in our clinic was based on anteroposterior radiographs, and sagittal and axial fracture patterns were not specifically described [5]. Moreover, even with the CT-based fracture classification recently proposed by many authors, the description of fracture patterns was still inadequate [6–11]. Nowadays, 3D CT was increasingly used in the clinic, which provided a great convenience for further understanding of tibial plateau fractures [12].

The fracture mapping technique was first proposed in scapular fractures by Armitage et al. [13]. This technique was subsequently applied to scaphoid fractures, distal radius fractures, pilon fractures, acetabular fractures, and many more fractures [14–19]. In recent years, fracture mapping and fracture heat maps have been reported for tibial plateau fractures [20–25]. However, all of these were two-dimensional fracture mapping limited within the articular surface. In tibial plateau fractures, focusing on the fracture pattern of the axial articular surface alone would be insufficient to help the surgeon plan the surgical approach. Therefore, 3D fracture mapping of TPFs needs to be investigated.

The main purpose of this study was to comprehensively demonstrate the fracture morphology of TPFs of different Schatzker types by mapping the fracture lines of a series of TPFs on a 3D model of the proximal tibia. Moreover, this study also associated Schatzker classification and ten-segment classification to analyze the injury frequencies of different segments and their variability. This study aimed to better assist surgeons in recognizing TPFs by presenting 3D fracture mapping and morphological characteristics.

## Materials and methods

### Patient sample

Patients with TPFs admitted to the Department of Orthopaedic Trauma of two level I trauma centres between January 2017 and December 2020 were collected. The inclusion criteria for this study were (1) age ≥ 18 years, (2) closed fracture, (3) AO/OTA classification type B and C fracture, and (4) complete pre-operative clinical and imaging data, and the exclusion criteria were (1) obsolete fracture, (2) pathological fracture, (3) substandard CT scans, and (4) multiple injuries of bone and joint. Ultimately, 346 patients were included in this study. These patients were numbered according to the time of consultation. All of the included patients’ casefiles and imaging files were completely collected and saved in their respective numbered folders by a specific researcher. In addition to epidemiological data (age and gender), the trauma mechanism was recorded.

### Fracture classifications

**Fig. 1:**
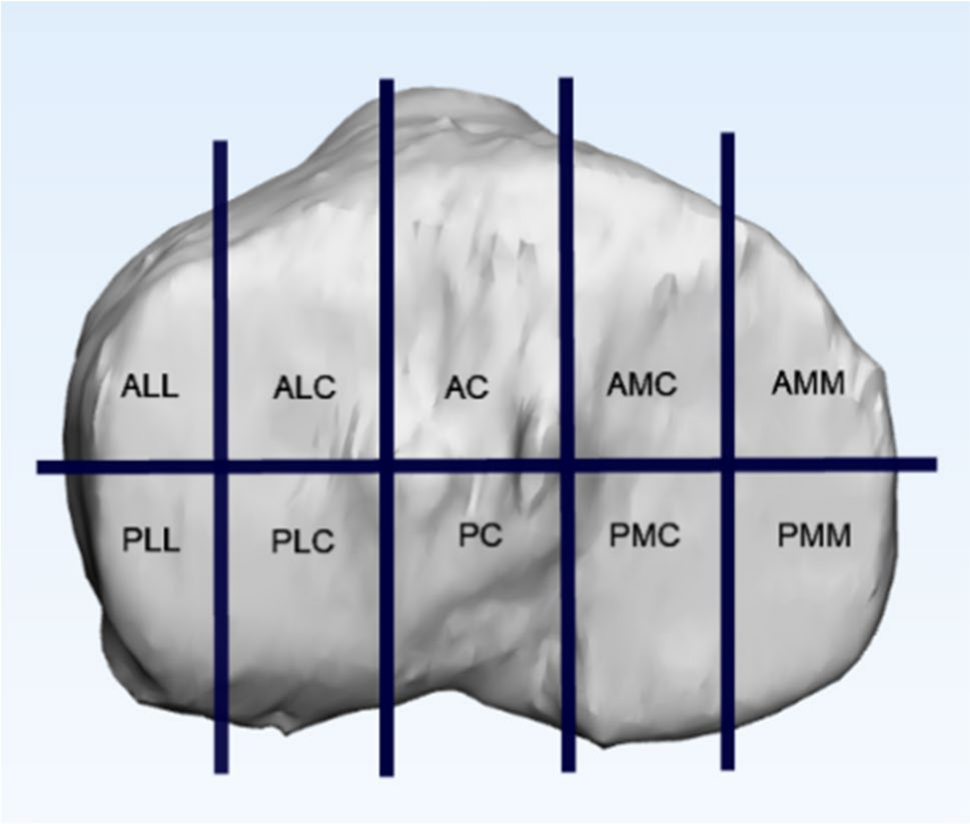
Ten-segment classification: AMM, antero-medio-medial; AMC, antero-medio-central; PMM, postero-medio-medial; PMC, postero-medio-central; AC, antero-central; PC, postero-central; ALL, antero-latero-lateral; ALC, antero-latero-central; PLL, posterolatero-lateral; PLC, postero-latero-central

Krause’s proposed ten-segment classification was based on pre-operative CT scans [8]. It divides the articular surface of the tibial plateau into anterior and posterior columns in the axial position. The anterior and posterior columns were further divided into five separate segments. In this way, the tibial plateau was divided into ten separate segments (Fig. 1).

Schatzker classification was proposed in the literature by Schatzker et al. in 1976 [5]. With subsequent refinement and analysis by authors, it had become one of the widely used and generally accepted classification systems for tibial plateau fractures [7]. Schatzker classified TPFs into six types: type I, split wedge of the lateral tibial plateau; type II, split wedge depression of the lateral tibial plateau; type III, pure depression of the lateral tibial plateau; type IV: split wedge of the medial tibial plateau; type V: bicondylar tibial plateau fracture, where there is continuity between the epiphysis and the diaphysis; type VI: bicondylar fracture with complete dissociation between the epiphysis and the diaphysis.

Imaging files of TPFs from all patients we collected were grouped according to Schatzker classification, and those that could not be typed were classified individually. At least three senior orthopedic surgeons in attendance who participated in this study jointly performed fracture classification on the imaging files of the patients. Moreover, at least one of the chief trauma orthopedic surgeons reexamines the imaging files for which classification has been completed. All data were categorized and sorted by one professional researcher.

### 3D fracture mapping

Export the pre-operative CT profiles of all patients who have been included in this study. The patients’ CT scan files were exported in Digital Imaging and Communication in Medicine (DICOM) format and were reconstructed in 3D using the Mimics 21.0 system (Materialise, Belgium). Simulated repositioning of all reconstructed fracture models was performed in the software. The repositioned 3D models were imported into 3-matic research 13.0 (Materialise, Belgium) software for rotation, mirror flip, and standardization of dimensions. This was done to position, overlap, and superimpose all fracture lines of the reconstructed models on a standard 3D model of the tibia. Thus, a series of standardized reconstruction models of TPFs were obtained.

**Fig. 2:**
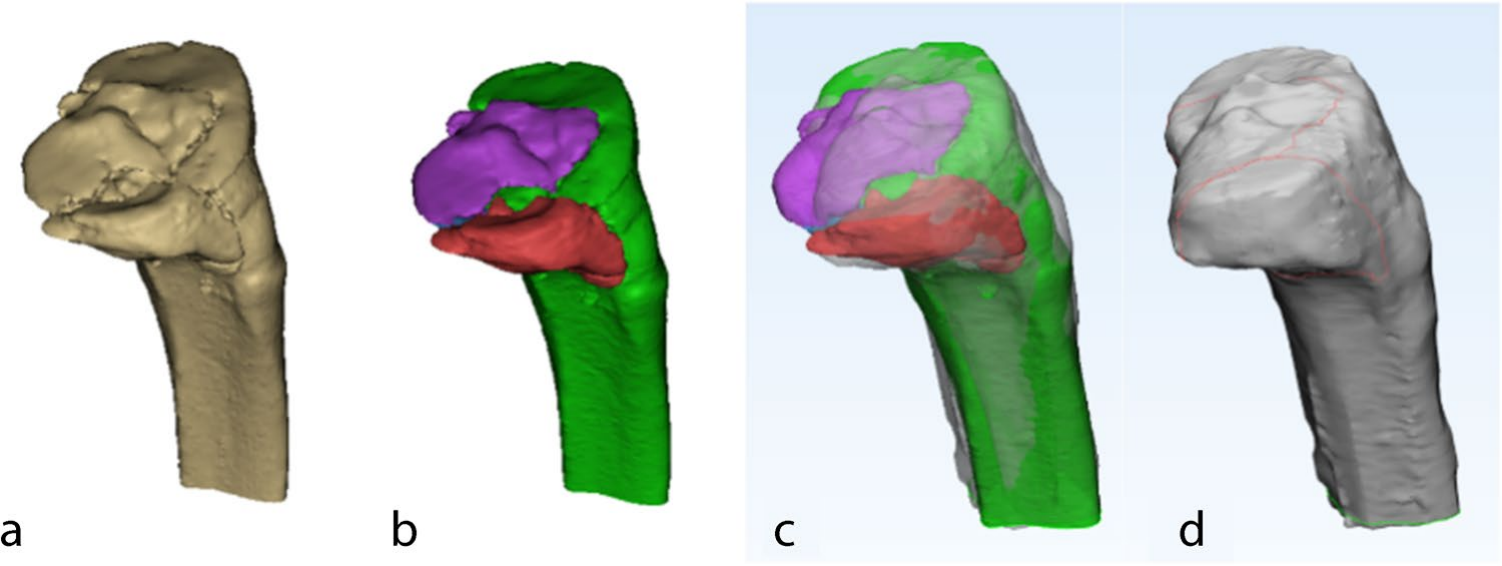
The method used for the mapping of tibial plateau. **a** Making 3D reconstruction of fractures. **b** Separation and repositioning of fracture blocks. **c** Matching 3D reconstructions with standard models. **d** Creating 3D fracture mapping

**Fig. 3:**
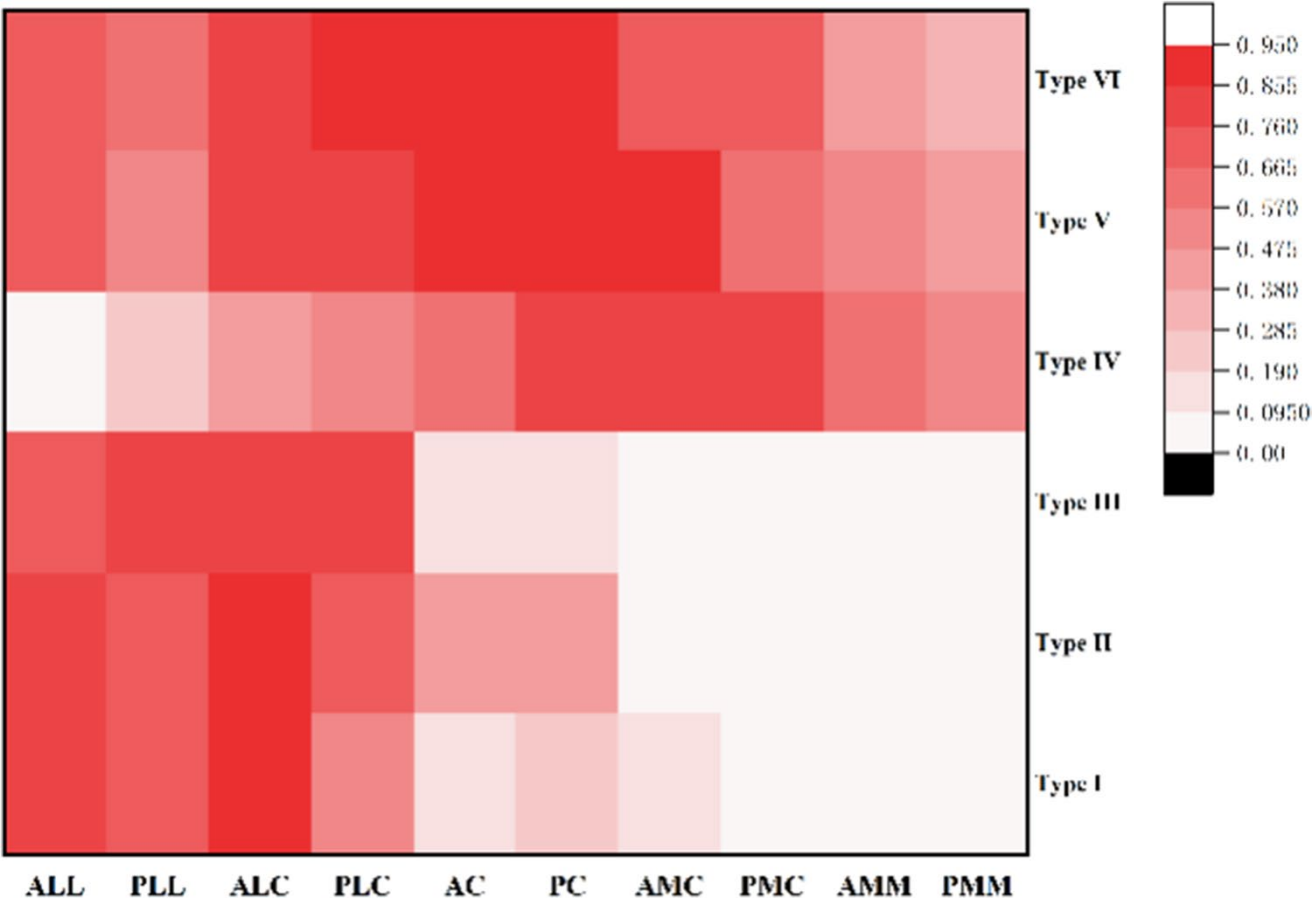
Frequency of different types of fractures at different segments

An adult male left tibia 3D CT reconstruction (30 years, no history of knee trauma) was selected as our standard proximal tibial model. Therefore, all patients with right TPFs in the study were required to have their fracture 3D reconstructions mirror-flipped. We set a series of localization areas for the standard proximal tibial model, including anatomical landmarks such as the anterior tibial ramus, Gerdy’s node, medial and lateral tibial condyle areas, intercondylar ramus, anterior and posterior intercondylar areas, and medial and lateral collateral ligament attachment points. Positioning correction of the fracture model to the standard model. Once anatomical marker alignment was obtained, fracture lines could be drawn using the curve create tool in 3-matic research. If there were deviations, use the curve edit tool to make 3D adjustments. Afterwards, each fracture line was renamed and noted with patient information and number for subsequent presentation (Figs. 2 and 3).

### Date analysis

The 3D fracture mapping obtained was merged according to the Schatzker classification in order to identify regularities of the corresponding fracture entities through the virtual overlay image. 3D fracture mapping were overlaid with ten-segment classification. The number of fracture lines in different segments was counted in order to analyze the injury rate.

Statistical analysis was carried out using SPSS software package version 26.0 (SPSS Inc., Chicago, IL). (1) Differences between categorical data were assessed by chi-square test. Comparisons between groups were tested using adjust *p*-values (Bonferroni method); (2) identification of the regularity of the corresponding fracture entities by 3D fracture mapping.

## Results

In total, 346 patients with TPFs were analyzed, including 204 male patients (59%) and 142 female patients (41%). The mean age of the patients was 47.3 years (44.8 years for male and 50.8 years for female). Using the Schatzker classification, we found 39 type I (11.27%), 103 type II (29.77%), 9 type III (2.60%), 71 type IV (20.52%), 52 type V (15.03%), 59 type VI (17.05%) fractures, and 13 others (3.76%). The characteristics of the patients are summarized in Table 1.

**Table 1:** Patient demographics (*N* = 346)

Variable	
Sex (no.[%])	
Male	204 (59)
Female	142 (41)
Mean age (range) (yr)	47.3 (18–82)
Side of injury (no.[%])	
Left	205 (59)
Right	141 (41)
Fracture distribution (no.[%])	
Type I	39 (11)
Type II	103 (30)
Type III	9 (3)
Type IV	71 (20)
Type V	52 (15)
Type VI	59 (17)
Others*	13 (4)
Injured segments (no.[%])	
ALL	211 (61)
ALC	257 (74)
AC	199 (58)
AMC	162 (47)
AMM	99 (29)
PLL	196 (57)
PLC	239 (69)
PC	224 (65)
PMC	145 (42)
PMM	86 (25)

^*^It is not possible to distinguish by Schatzker classification

**Fig. 4:**
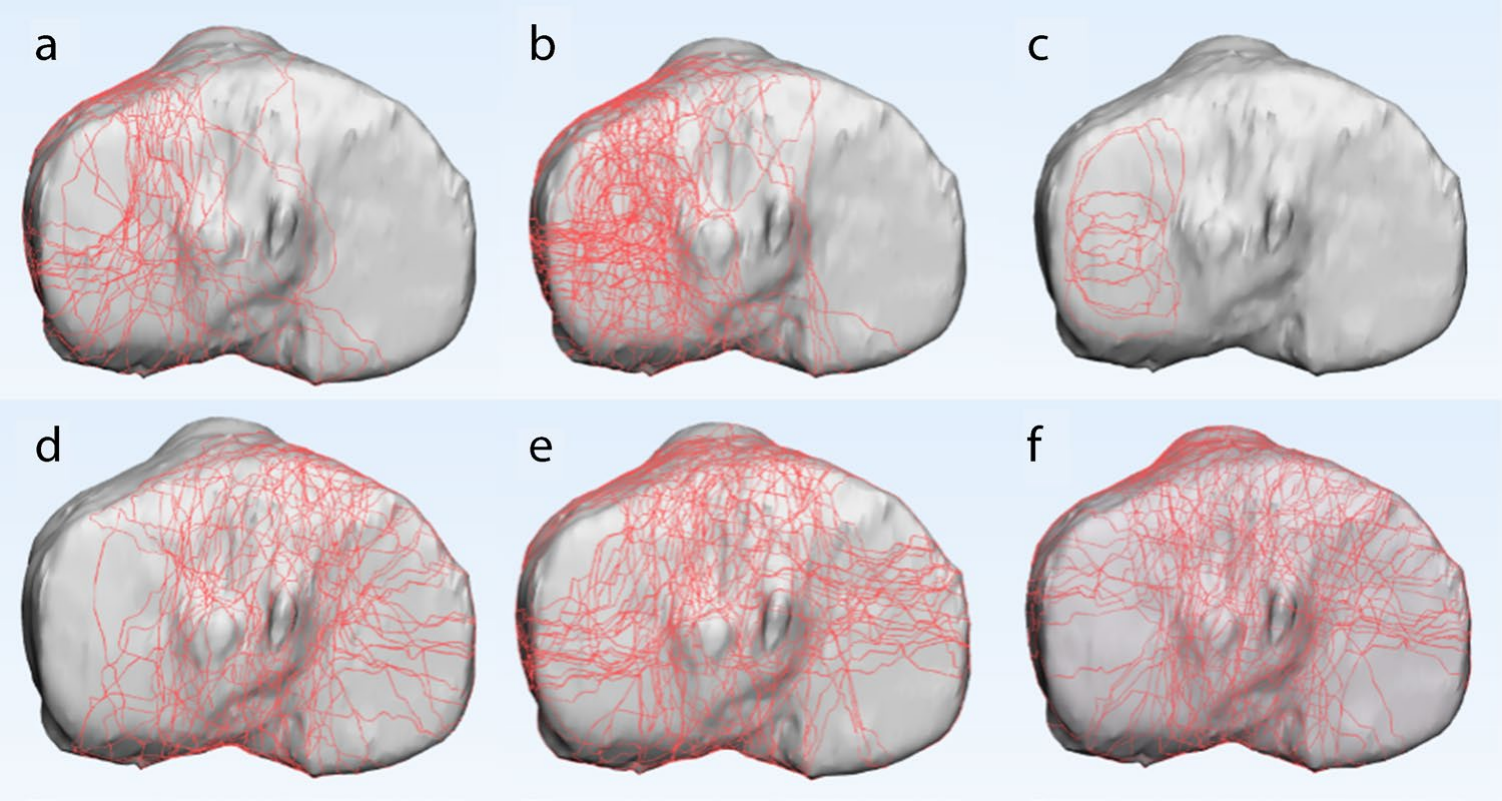
Schatzker type I–VI 3D fracture mapping in axial view. **a** and **b** represent types I–VI, respectively

**Fig. 5:**
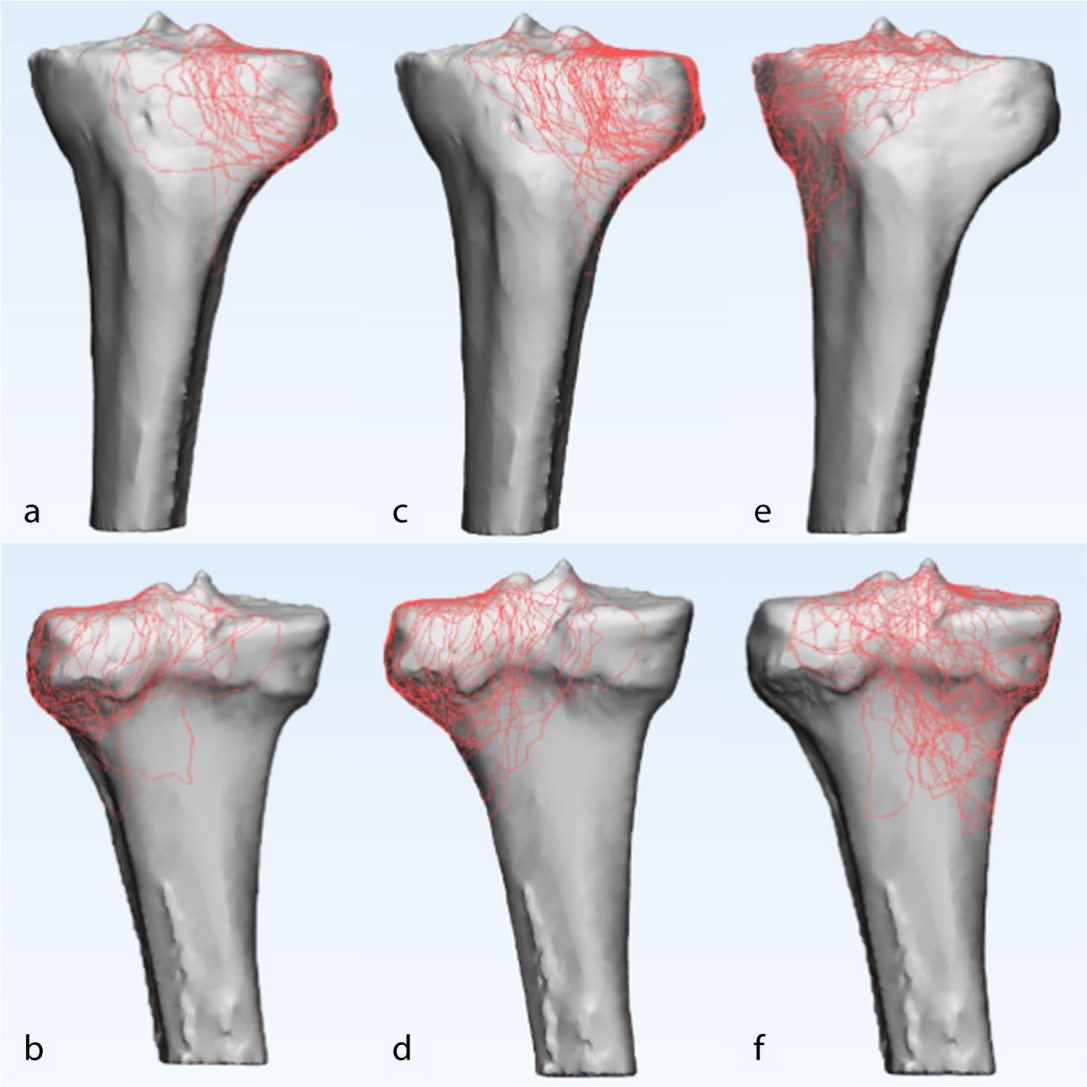
Schatzker type I, II, and IV 3D fracture mapping in coronal view. The upper images (**a**, **c**, **e**) are anterior coronal views; the lower images (**b**, **d**, **f**) are posterior coronal views. **a** and **b** represent type I; **c** and **d** represent type II; **e** and **f** represent type IV

**Fig. 6:**
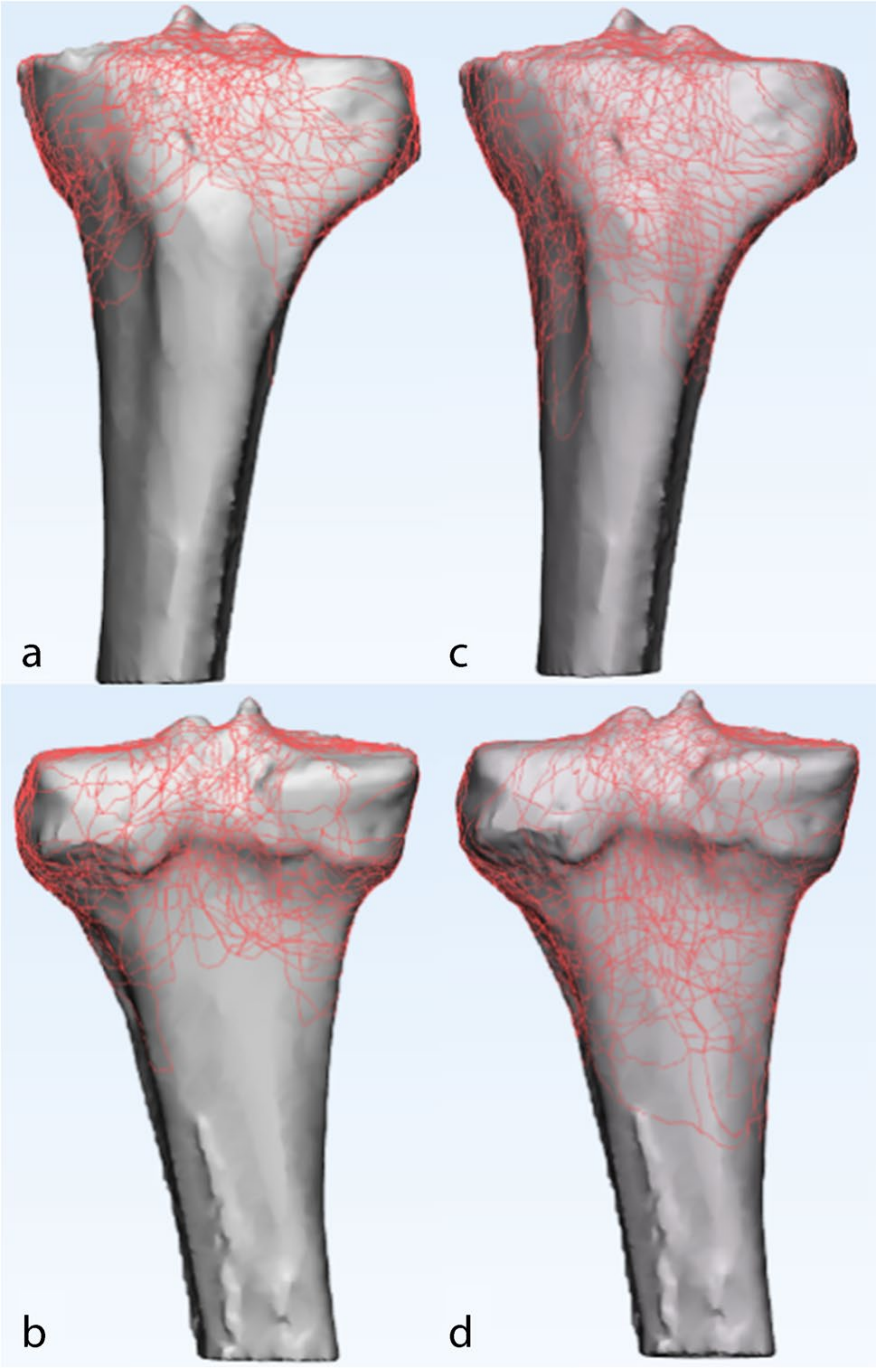
Schatzker type V and VI 3D fracture mapping in coronal view. The upper images (**a**, **c**) are anterior coronal views; the lower images (**b**, **d**) are posterior coronal views. **a** and **b** represent type V; **c** and **d** represent type VI

**Fig. 7:**
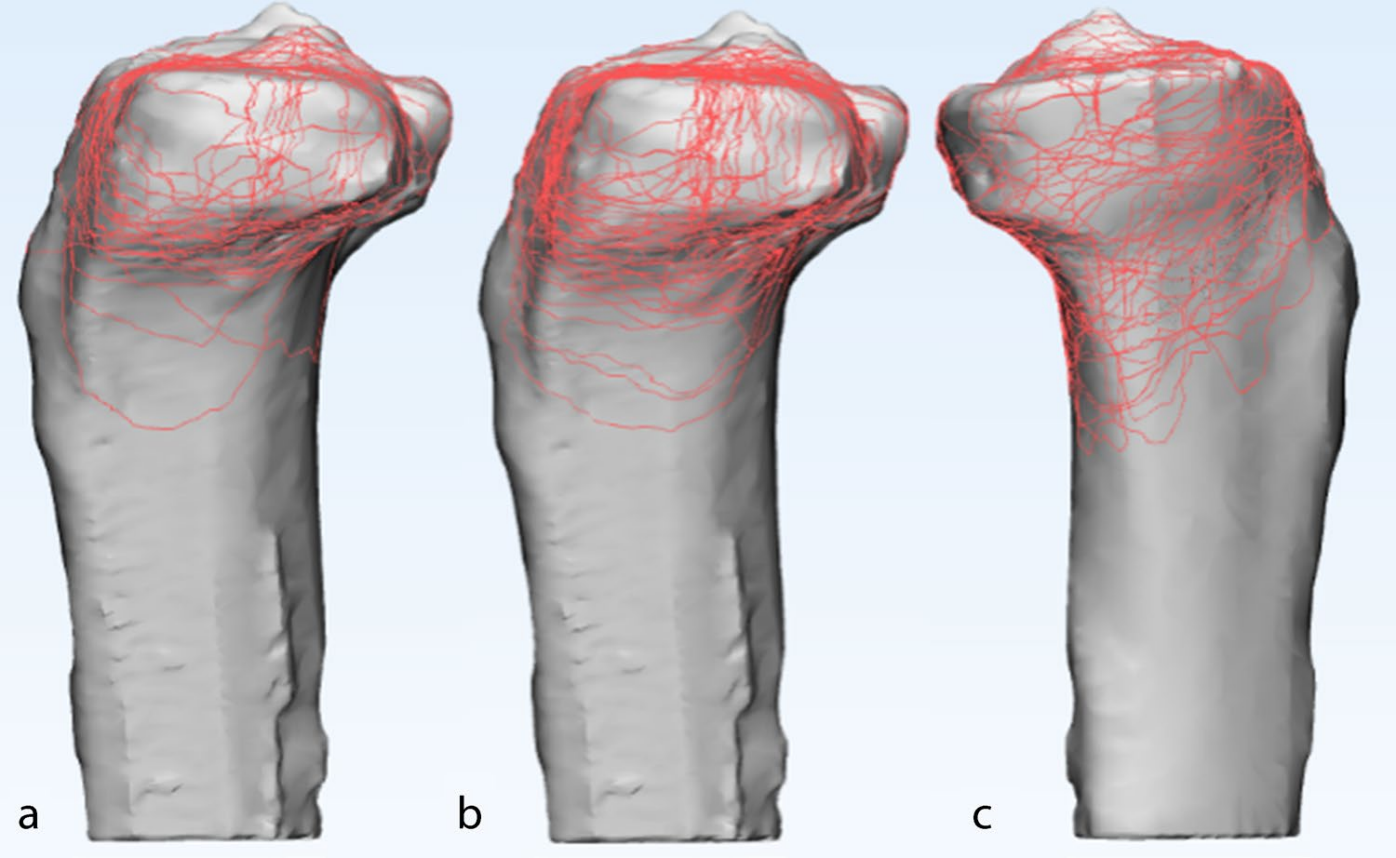
Schatzker type I, II, and IV 3D fracture mapping in sagittal view. **a** represents the lateral sagittal view of type I. **b** represents the lateral sagittal view of type II. **c** represents the medial sagittal view of type IV

**Fig. 8:**
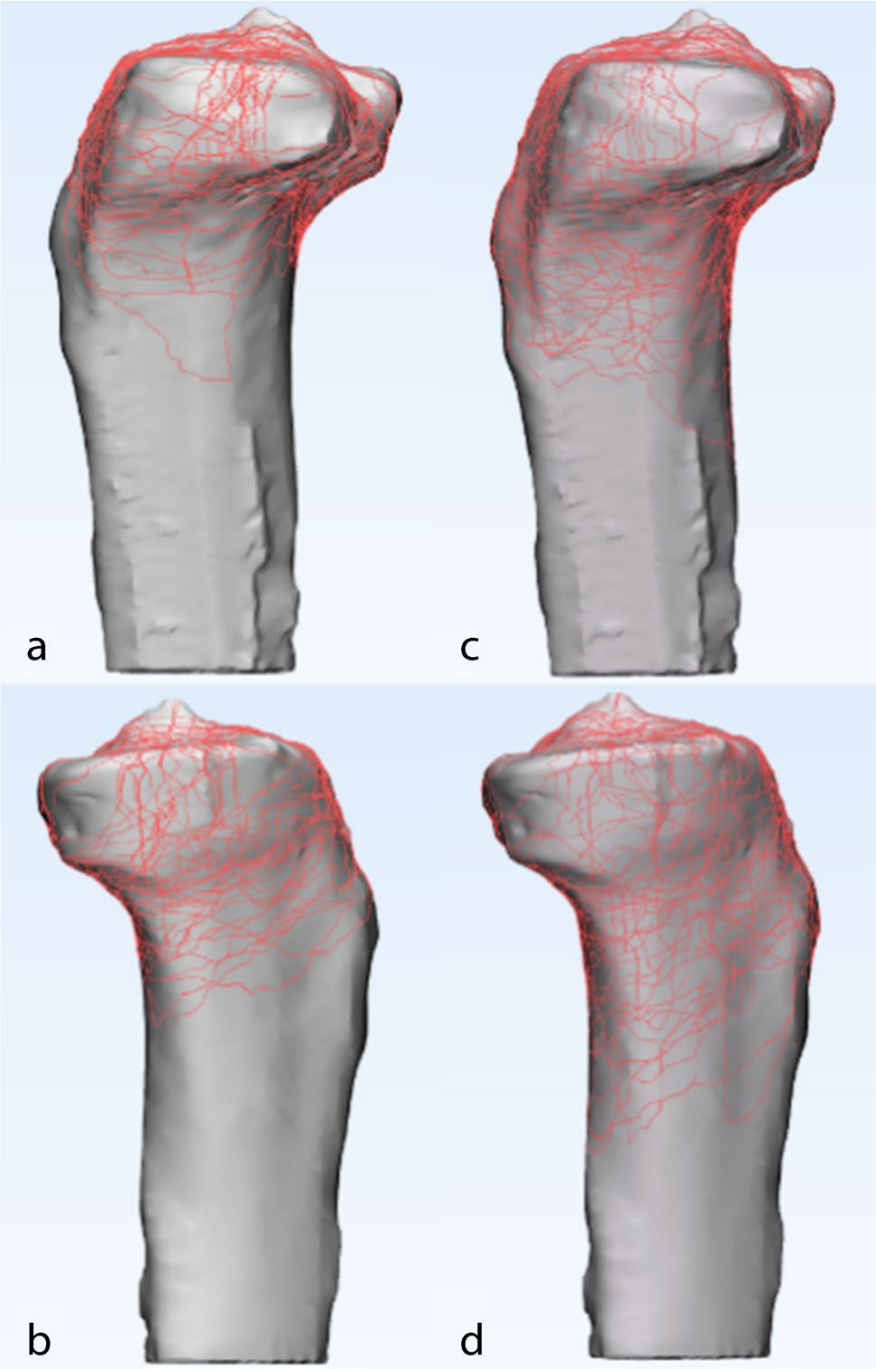
Schatzker type V and VI 3D fracture mapping in sagittal view. The upper images (**a**, **c**) are lateral sagittal views; the lower images (**b**, **d**) are medial sagittal views. **a** and **b** represent type V; **c** and **d** represent type VI

**Fig. 9:**
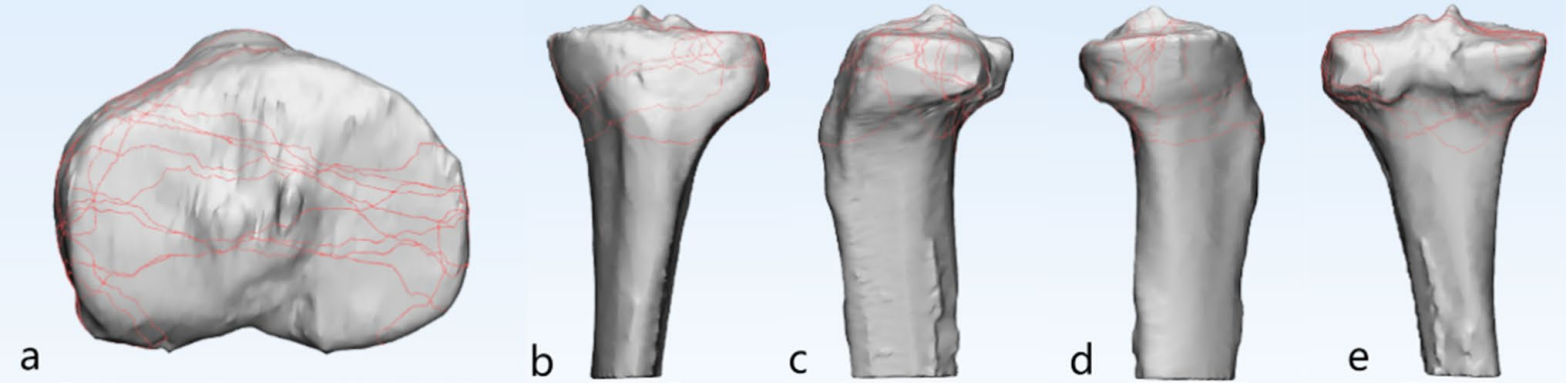
Special type 3D fracture mapping. **a**–**e** represent axial view, anterior coronal view, lateral sagittal view, medial sagittal view, and posterior coronal view of special fracture types, respectively

The 3D fracture mappings for the different Schatzker types are shown in Figs. 4, 5, 6, 7 and 8. For special types that could not be distinguished by Schatzker classification, we unified them in a 3D model (see Fig. 9). The 3D fracture mappings of the different types were matched to the ten-segment classification and the frequency of occurrence of the different segments was counted, and the results can be seen in Table 2 and Fig. 3. The segmental frequency of TPFs injury was counted for all 3D fracture mapping overlay models matched with ten-segment classification, as shown in Table 2.

**Table 2:** Results of injury frequency and intergroup variability of different segments under Schatzker classification

Schatzker	Ten segments									
	ALL	ALC	AC	AMC	AMM	PLL	PLC	PC	PMC	PMM
Type I	0.82^a^	0.90^a^	0.15^a^	0.10^a^	0^a^	0.69^a^	0.56^a,b^	0.28^a^	0.08^a^	0.03^a^
Type II	0.83^a^	0.86^a^	0.42^b^	0.08^a^	0^a^	0.71^a^	0.72^a,b,c^	0.43^a^	0.08^a^	0.04^a^
Type III	0.67^a^	0.78^a,b^	0.11^a,b^	0^a^	0^a,b^	0.78^a^	0.78^a,b,c^	0.11^a^	0^a^	0^a,b^
Type IV	0.01^b^	0.41^b^	0.61^b^	0.76^b^	0.59^c^	0.25^b^	0.51^b^	0.83^b^	0.79^b^	0.52^c^
Type V	0.73^a^	0.81^a^	0.88^c^	0.88^b^	0.48^b,c^	0.56^a^	0.83^a,c^	0.90^b^	0.65^b^	0.42^b,c^
Type VI	0.73^a^	0.80^a^	0.90^c^	0.73^b^	0.41^b,c^	0.64^a^	0.90^c^	0.95^b^	0.75^b^	0.32^b,c^
*P*-value	*P* < 0.01	*P* < 0.01	*P* < 0.01	*P* < 0.01	*P* < 0.01	*P* < 0.01	*P* < 0.01	*P* < 0.01	*P* < 0.01	*P* < 0.01

If the subscript letters are different between any two groups, it means that the difference between these two groups is statistically significant, *P* < 0.05

### Schatzker classification 3D fracture mapping

#### Axial view

Schatzker type I: Most TPFs fracture lines extend from the anterior tibial tuberosity and its lateral aspect toward the posterior lateral condyle. In addition, some of the fracture line extended from the anterior border area of the fibular head toward the posterior medial condyle. A few fracture lines extend longitudinally across the intercondylar eminence; Schatzker type II: most fracture lines were located on the lateral side of the tibial intercondylar eminence. Fracture lines extended over the lateral aspect of the anterior tibial tuberosity, the anterior border area of fibular head, and the posterior aspect of tibial intercondylar eminence formed a curved dense area; Schatzker type III: fracture lines in the area of collapse were irregularly oval in the lateral condyle; Schatzker type IV: fracture lines were widely scattered on medial and lateral side of the intercondylar eminence. A portion of the fracture lines extended to the posterior lateral aspect; Schatzker type V: fracture lines were distributed in a cross pattern on the articular surface of plateau. The medial and lateral condylar regions were present with intact areas not encroached by fracture lines; Schatzker type VI: fracture lines were also distributed in a cross-like pattern, and the articular surfaces were extensively invaded.

#### Coronal view

Schatzker type I and type II: Fracture lines were distributed in the anterior coronal plane mainly in the region where the lateral condyle migrated from the tibial shaft. In the posterior coronal plane, fracture lines extended downward from the articular surface complexly and disorderly; Schatzker type IV: fracture lines in the anterior and posterior coronal planes were distributed disorderly in a spider web pattern, with no obvious convergence areas; Schatzker type V: in the anterior coronal plane, fracture lines ran superiorly to the tibial anterior tuberosity to both sides and extended posteriorly. Only few fracture lines invaded the tibial anterior tuberosity. In the posterior coronal plane, fracture lines extend mainly downward from the posterior intercondylar area; Schatzker type VI: in the anterior coronal plane, most fracture lines invaded the tibial anterior tuberosity and below it, extending posteriorly to the sides.

#### Sagittal view

Schatzker type I: fracture lines were scattered in the lateral sagittal plane. The lowest point of fracture lines were mostly located in the lateral sagittal view; Schatzker type II: fracture lines formed a strip-type high-density area in the central region of the lateral condyle. The lowest point of fracture lines was mostly located in the lateral sagittal view; Schatzker type IV: fracture lines presented a disordered reticular distribution in the medial sagittal view, with no obvious convergence area; Schatzker type V and Schatzker type VI: fracture lines distributed in the lateral sagittal view as type II and in the medial sagittal view as type IV.

### Special types 3D fracture mapping

In total, 13 cases of TPFs could not be accurately distinguished by Schatzker classification. Among them, there were four cases of anterior coronal fractures. In three of these cases, fracture lines extended from the lateral aspect of the tibial anterior tuberosity to the medial condyle centrally, and one of these cases had a posterior lateral condylar avulsion fracture. In the other case, the fracture line extended from the medial aspect of the tibial anterior tuberosity to the central aspect of the lateral condyle. There were five cases of posterior coronal plane fractures. The lowest point of fracture lines in these cases was located in the posterior coronal view or medial sagittal view. There were four cases of tibial plateau edge avulsion fractures, three of which were located in the lateral condyle and one in the medial condyle.

## Discussion

Fracture mapping was a very visual way to show the fracture pattern [13, 14]. In our study, we used this technique to present 3D fracture mapping of TPFs in 346 cases under different Schatzker classification, as well as some special types of fractures that could not be distinguished by Schatzker classification. In this way, to better demonstrate fracture injury patterns and morphological characteristics. Also, the incidence of different types of fracture injury segments was counted. We found significant variability in the morphology and the distribution pattern of fracture lines among the different Schatzker types (Table 2).

Our study found a significantly higher number of lateral fracture lines (881/1765) than medial (474/1765) in the axial view. The number of fracture lines in the intercondylar eminence areas (410/1765) was inferior to the above two. The areas of dense bone fracture lines were mainly located in the ALC and PLC segments (74%, 69%). In addition to fracture lines extending longitudinally along the ALC and PLC segments, fracture lines also form a high-density pooling area between the intercondylar eminence and the anterior edge of the fibular head. In the anterior coronal view, the high-density area of fracture lines was mainly located lateral to the anterior tibial tuberosity and extended downward and posteriorly. Meanwhile, in the posterior coronal plane, fracture lines were mainly concentrated in the medial–lateral condyle migration to the depression posterior of the intercondylar eminence, i.e., the PC segment. In the lateral sagittal view, fracture lines were mainly converging in the central zone of the lateral condyle, i.e., the area where the ALL and PLL segments meet in the sagittal plane. In the medial sagittal view, fracture lines were distributed in a complex dense grid, and the researchers observed no obvious convergence areas.

3D fracture mapping showed unique features of fracture lines in different fracture types. We believed that the phenomenon of significant local convergence of fracture lines is not only related to injury mechanisms [26]. The proximal tibial bone microarchitecture was also considered to have an impact. Krause et al. found that the tibial plateau bone microarchitecture was unevenly distributed between healthy people and patients with osteoporosis; these findings result in different vulnerability to mechanical load [27].

The study by Wang et al. found that valgus force often produced compression in the lateral plateau and medial plateau fractures with the fracture line lateral to the intercondylar spines [28]. In contrast, varus force often produced medial plateau fractures with the fracture line medial to the intercondylar spines or within the intercondylar spines. Our study found that fracture lines of Schatzker type II were mainly concentrated in ALL, ALC, PLL, and PLC segments (0.573, 0.631, 0.495, 0.504). It was more likely that fracture lines of Schatzker type IV violated AC and PC segments of the intercondylar eminence areas (0.437, 0.592). Moreover, the medial condyle was usually completely split and the articular surface of the fracture fragments was not easily collapsed.

Schatzker proposed TPFs classification in 1976 based on traditional anterior–posterior plain films [5]. It can distinguish most of the fracture patterns, but cannot distinguish the different characteristics of each sub-type, which needs to be complemented by 3D fracture mapping. Based on our 3D fracture line mapping, we show the morphological characteristics of the different Schatzker classifications in axial and sagittal positions, which enriches the orthopedic surgeon’s knowledge of the classification and facilitates the delineation of more sub-types. Moreover, we found that some fracture patterns cannot be well distinguished by the Schatzker classification in clinical practice. In our study, there were 13 cases that could not be distinguished using Schatzker classification. And, only nine cases (2.6%) of Schatzker type III were found, less than the specific types of TPFs (3.8%).

Samsami et al. found that coronal plane fracture lines significantly affect the mechanical response of tibial implant structures, especially medially, through their study [29]. Yet, this type of fracture models was not distinguishable by Schatzker classification. In our study, simple coronal plane fractures were accounted for 2.3% of all patients. Although it is a small percentage of all TPFs types, it did not make it negligible. Coronal plane fractures were caused by vertical shear violence. The mechanisms of injury were mostly hyperextension or hyperflexion [25, 26, 30]. Especially for PMC and PMM segments, they showed a higher instability and complexity of treatment.

Molenaars et al., who mapped tibial plateau fractures in 127 patients in 2015, analyzed and identified four main fracture characteristics [23, 24]: (1) the lateral split fragment with or without comminution, (2) the posteromedial fragment, (3) the tibial tubercle fragment, and (4) a zone of comminution including the tibial spine. They concluded that by distinguishing between the four specific fracture patterns described above would improve inter-observer agreement more than by classifying fractures specifically. McGonagle et al. mapped fracture lines in 261 patients with TPFs in 2019 [21]. They concluded that TPFs followed relatively consistent fracture patterns. Moreover, most of the lateral and medial plateau fracture lines were located in the sagittal plane, but medial plateau fracture lines were more variable. Kerschbaum et al. mapped TPFs lines in axial view after typing 278 TPFs by AO/OTA and Schatzker classification in 2021 [20]. They confirmed the importance of pre-operative CT scans. Also, they found that fracture patterns and lines varied considerably even within each fracture subgroup. This was similar to our study findings.

Although there were some similarities between the study of Kerschbaum and ours, our study presented a more comprehensive view of the three-dimensional morphological characteristics of the different types of fractures. We distinguished six Schatzker types of fracture lines as well as specific types of TPFs fracture lines and presented them using a 3D model. This was very intuitive for the orthopsedic surgeons.

With the continuous development of computed tomography (CT), the description of fracture patterns has risen from two-dimensional to three-dimensional. Preoperative CT scanning and 3D reconstruction had been widely promoted in TPFs. Therefore, the emergence of CT-based classification tools had become an inevitable trend. Many authors suggested that pre-operative evaluation of TPFs using 3D CT would be more accurate in developing surgical strategies [7, 12, 31]. In recent years, several authors had proposed CT-based classification tools for tibial plateau fractures [6–11]. Most of them used the concept of columns and segments to describe the axial articular surfaces and the bone blocks beneath the articular surfaces.

The ten-segment classification was proposed by Krause et al. He used axial CT scanning to identify fracture segments at 3 cm below the articular surfaces and divided the axial articular surfaces into 10 segments [8]. This classification demonstrated higher inter-observer agreement when using 3D CT in another study by our team [32]. We found that the frequency of injury to the ALC was highest (0.719) and the PMM was lowest (0.238) in all fracture lines. In addition to this, the frequency of PLC (0.679) injuries was also very high, indicating a high probability of posterior lateral condyle injury in lateral TPFs. Also the anterior–posterior intercondylar eminence segment damage represented by AC (0.555) and PC (0.630) suggests that for TPFs one must be aware of the risk of combined cruciate ligament injury.

Injuries in different segments or regions influenced the choice of surgical approach [29, 33]. For TPFs, the literature usually reports anterolateral or medial approaches. However, some regional injuries, like the postero-lateral and postero-medial bone blocks, were not able to achieve sufficient fixation through these traditional approaches [34]. Many new surgical approaches were reported [35–37]. A study by Krause et al. suggested a significant difference in tibial plateau articular surface exposure with different surgical approaches [38]. The surgeon was required to adequately assess the area of fracture damage and observe the fracture line alignment. The selection of an appropriate surgical approach as a means of minimizing trauma to the patient during surgery was one purpose of our study. We found that for Schatzker type I and type II, there are obvious characteristics of fracture line distribution. According to the main segment of the fracture, it can be distinguished as anterolateral and posterolateral fractures. In anterolateral Schatzker type I and II fractures, the lowest point of the fracture fragment is mainly located in the lateral sagittal plane, whereas the lowest point of the posterior lateral fracture is mostly located in the posterior coronal plane. This can be a new subtype of Schatzker type I and II and importantly influences the choice of surgical approach.

Three-dimensional reconstruction technique demonstrated the fracture pattern more clearly [19, 25, 39]. This technique brought great convenience in fracture morphology, location, and surgical approach selection. Since joint incongruity often involves three-dimensional displacement in multiple planes (e.g., gaps and steps), only 3D CT can provide a direct demonstration of the fracture fragments, including the anatomical position of the lower part of the fracture fragment, the separation of the articular surface, and the degree of collapse. Using 3D reconstruction models to create 3D fracture mapping allowed the orthopedic surgeon to view fracture patterns in all directions.

The study by Yao et al. performed 3D reconstruction of CT scans and mapped fractures in 759 TPFs patients, and finally generated 3D heat maps [11, 25]. They constructed a fracture classification of four columns and nine segments by different heat zones. Their study found that hot zones of TPFs were mostly found in the lateral condyle and intercondylar eminences, and less in the medial condyle. Tibial plateau fracture lines were seen mostly in the displaced areas between the different segments. This was more consistent with our results. However, their classification described only the axial articular surface of tibial plateau, ignoring the morphological features of fractures and injury mechanisms. The aim of our study was to demonstrate the specific three-dimensional characteristics of different types of fractures.

Some shortcomings of this study have to be considered. First, we used very strict inclusion and exclusion criteria when collecting patient history data, which forced us to discard many cases. We excluded at least six cases of bilateral TPFs and a proportion of cases with multiple osteoarticular injuries. Second, injury mechanisms were not accounted for as a classification criterion. There is a strong correlation between the occurrence and morphology of fractures and injury mechanisms. We considered the 3D fracture mapping to be a very good description of the different types of TPFs. However, it required further introduction of morphological parameters of fracture blocks for statistical and analytical purposes.

In conclusion, this study mainly showed the morphology of different types of TPFs by 3D fracture mapping. By superimposing Schatzker with ten-segment classification, the variability of segments with different types of fracture injuries was found. According to 3D fracture mapping, there were distinct distribution characteristics of fracture lines in different types of TPFs, which had great significance for clinical practice.
